# Monitoring Lung Aeration during Respiratory Support in Preterm Infants at Birth

**DOI:** 10.1371/journal.pone.0102729

**Published:** 2014-07-16

**Authors:** Liane J. Kang, Po-Yin Cheung, Gerhard Pichler, Megan O’Reilly, Khalid Aziz, Georg M. Schmölzer

**Affiliations:** 1 Division of Neonatology, Department of Pediatrics, University of Alberta, Edmonton, Canada; 2 Neonatal Research Unit, Royal Alexandra Hospital, Alberta Health Services, Edmonton, Canada; 3 Division of Neonatology, Department of Pediatrics, Medical University, Graz, Austria; Icahn School of Medicine at Mount Sinai, Argentina

## Abstract

**Background:**

If infants fail to initiate spontaneous breathing, resuscitation guidelines recommend respiratory support with positive pressure ventilation (PPV). The purpose of PPV is to establish functional residual capacity and deliver an adequate tidal volume (V_T_) to achieve gas exchange.

**Objective:**

The aim of our pilot study was to measure changes in exhaled carbon dioxide (ECO_2_), V_T_, and rate of carbon dioxide elimination (VCO_2_) to assess lung aeration in preterm infants requiring respiratory support immediately after birth.

**Method:**

A prospective observational study was performed between March and July 2013. Infants born at <37 weeks gestational age who received continuous positive airway pressure (CPAP) or PPV immediately after birth had V_T_ delivery and ECO_2_ continuously recorded using a sensor attached to the facemask.

**Results:**

Fifty-one preterm infants (mean (SD) gestational age 29 (3) weeks and birth weight 1425 (592 g)) receiving respiratory support in the delivery room were included. Infants in the CPAP group (n = 31) had higher ECO_2_ values during the first 10 min after birth compared to infants receiving PPV (n = 20) (ranging between 18–30 vs. 13–18 mmHg, p<0.05, respectively). At 10 min no significant difference in ECO_2_ values was observed. V_T_ was lower in the CPAP group compared to the PPV group over the first 10 min ranging between 5.2–6.6 vs. and 7.2–11.3 mL/kg (p<0.05), respectively.

**Conclusions:**

Immediately after birth, spontaneously breathing preterm infants supported via CPAP achieved better lung aeration compared to infants requiring PPV. PPV guided by V_T_ and ECO_2_ potentially optimize lung aeration without excessive V_T_ administered.

## Introduction

In utero the airways are liquid-filled and the lungs take no part in gas exchange, which occurs across the placenta [1]. At birth lung liquid is cleared to allow air entry and establishment of a functional residual capacity (FRC) [1], [2]. Although most preterm infants breathe spontaneously at birth [3], many require continuous positive airway pressure (CPAP) to support lung aeration [4]. Indeed, extremely preterm infants have immature lungs that are surfactant deficient, partially liquid filled, and prone to collapse at end expiration, and often require respiratory support at birth [2]. If infants fail to initiate spontaneous breathing, neonatal resuscitation guidelines recommend positive pressure ventilation (PPV) [5]. The purpose of PPV is to establish functional residual capacity, deliver an *adequate* tidal volume (V_T_) to establish a functional residual capacity and facilitate gas exchange while minimizing lung injury [6]. Currently, heart rate (HR) and percutaneous oxygen saturation are used to assess if adequate ventilation occurred [5]. However, an objective assessment tool is lacking. *Hooper et al* recently described that exhaled CO_2_ (ECO_2_) can be used to assess degree of lung aeration [7]. Furthermore, a better lung aeration will initiate greater removal of CO_2_ during neonatal transition. To our knowledge, there is no information available regarding the changes in lung aeration after birth in preterm infants who are given CPAP or PPV as respiratory support at birth. The aim of this pilot study was to examine ECO_2_ and V_T_ changes in preterm infants either supported by CPAP or requiring PPV within the first 10 minutes after birth. In addition, we examined the rate of carbon dioxide elimination (VCO_2_) and minute ventilation. We also report the associated changes in hemodynamic and cerebral oxygenation parameters, which were recorded during the resuscitation of these preterm infants.

## Methods

This study was carried out at The Royal Alexandra Hospital, Edmonton, a tertiary perinatal center admitting more than 350 infants with a birth weight of <1500 g to the neonatal nursery annually. The Royal Alexandra Hospital Research Committee and Health Ethics Research Board, University of Alberta approved the study and parental consent was obtained to use the recordings. Infants <37 weeks post menstrual age and who were judged clinically to have inadequate breathing in the first minutes after birth were eligible for the trial. Infants were excluded if there was uncertainty about their gestational age or if they had a congenital abnormality that might adversely affect their breathing. Between March and July 2013, 70 deliveries of infants <37 weeks gestation were attended by the research team in addition to the Resuscitation-Stabilization-Triage team (usually a neonatal nurse, neonatal respiratory therapist, neonatal nurse practitioner, and neonatal fellow). The research team was not involved in the clinical care of the infants.

### Local resuscitation protocol

Cord clamping was routinely performed for 60 sec after delivery unless the obstetric team noted contraindications (e.g. bradycardia, apnea or antepartum hemorrhage and clamped earlier. If respiratory support was needed, it was started using air in babies >28 weeks gestation and using 30% oxygen if <28 weeks. Infants received CPAP and PPV via an appropriately sized round silicone face mask (Fisher & Paykel Healthcare, Auckland, New Zealand). Respiratory support was provided with a T-piece (Giraffe Warmer, GE Health Care, Burnaby, Canada) – a continuous flow, pressure-limited device with a built-in manometer and a positive end expiratory pressure (PEEP) valve. The default settings used were a gas flow of 8 L/min, peak inflation pressure (PIP) of 24 cm H_2_O and PEEP of 6 cm H_2_O. Local protocol dictated predefined intubation criteria if chest compressions were required, HR remained <100 despite 60 sec of PPV, or prolonged PPV of >10 min. The clinical team adjusted PIP, PEEP or fraction of inspired oxygen according to the infant’s need. All practitioners attending deliveries were trained in the use of equipment and in the Neonatal Resuscitation Program resuscitation protocol. In addition, all members of the neonatal resuscitation team are trained to use V_T_ and ECO_2_ monitoring and have experience and education with MRSOPA [8].

### Monitoring systems

Gas flow, V_T_, airway pressure, and ECO_2_ were continuously measured with the NM3 cardiopulmonary management monitor (NM3, Philips Healthcare, Electronics Ltd., Markham, ON, Canada). Airway pressure and gas flow were measured using fixed orifice differential pressure pneumotachometer. The NM3 calculates V_T_ by integrating the flow signal, the minute ventilation (MV) by integrating V_T_ and respiratory rate, and ECO_2_ using a non-dispersive mainstream infrared absorption technique. ECO_2_-value represents the peak value ( = end-tidal value) during expiration. In addition, the NM3 also measures carbon dioxide elimination (VCO_2)_. According to the manufacturer the accuracy for the gas flow is ±0.125 L/min and for ECO_2_ ±2 mmHg and the dead space of the flow sensor is ∼1mL.

IntelliVue MP50 (Philips Healthcare, Philips Electronics Ltd., Markham, ON, Canada) monitors were used to continuously record HR, arterial oxygen saturation, and blood pressure. A Masimo Radical pulse oximeter (Masimo Corporation, Irvine CA, USA) probe set at maximum sensitivity and two-second averaging was placed around the infant’s right wrist to measure oxygen saturation [9]. HR was measured using three Micro-Premie Leads (Vermed, Bellows Falls, VT, USA) and blood pressure using a non-invasive blood pressure cuff of appropriate size on the left upper arm. The left upper arm was chosen to avoid interference with the pulse oximetry measurements.

An Invos Cerebral/Somatic Oximeter Monitor (Invos 5100, Somanetics Corp., Troy, MI, USA) with the neonatal sensor was used to measure cerebral regional tissue oxygen saturation (crSO_2_). The transducer contains a single light emitting diode and two sensors that measure pooled hemoglobin saturation within two different tissue paths. The Invos Cerebral/Somatic Oximeter Monitor calculates the crSO_2_, which is expressed as the percentage of oxygenated hemoglobin (oxygenated hemoglobin/total hemoglobin). The transducer was positioned on the left fronto-parietal forehead and secured with a wrap in each infant, regardless of mode of delivery. The clinical team was able to observe all monitors; only the alarms at the NM3 and Invos Cerebral/Somatic Oximeter Monitor were disabled to avoid distraction of the clinical staff by research measurements.

### Data collection

Data collection started immediately after the baby was transferred to the resuscitation unit. In infants receiving delayed cord clamping (n = 29) data collection started around 90 sec after birth and infants without delayed cord clamping (n = 22) data collection started at around 30 sec after birth. All variables were stored continuously in a multichannel system “alpha-trace digital MM” (B.E.S.T. Medical Systems, Austria) for subsequent analysis. Values of gas flow, V_T_, airway pressure, and ECO_2_ were recorded at 200 Hz, arterial and regional oxygen saturation, and HR were stored every second, and the sample rate of crSO_2_ was 8 seconds (0.13 Hz). HR, oxygen saturation, oxygen requirements, and crSO_2_ were recorded for the first 30 min after birth. Blood pressure was measured every minute for the first 15 minutes, at 20, 25, and 30 min.

### Statistical analysis

Demographics of study infants were recorded. A breath-by-breath analysis of airway pressure, gas flow, V_T_, and ECO_2_ was performed and expired V_T_, airway pressure, gas flow, and ECO_2_ of all breaths and inflations were measured. In the PPV group respiratory parameters measured for spontaneous breaths and inflations were recorded. Mask leak was corrected for body temperature, pressure and water vapor saturation using a standardized equation. Spontaneous breaths and inflations with a mask leak >30% were excluded from further analysis as these could underestimate the expired V_T_ and ECO_2_ values. The data are presented as mean±standard deviation (±SD) for normally distributed continuous variables and median (interquartile range (IQR)) when the distribution was skewed. For all respiratory parameters, the median value for each infant was calculated first and then either the mean or median of the median calculated for parametric and non-parametric data, respectively. Data were compared using Student’s t-test for parametric and Mann-Whitney U test for nonparametric comparisons of continuous variables, and χ2 for categorical variables. P-values are 2-sided and p<0.05 was considered statistically significant. Statistical analyses were performed with Stata (Intercooled 10, Statacorp, Texas, USA).

## Results

A total of 51 preterm infants (31 received CPAP and 20 received PPV) were included in the study. 19 infants did not require respiratory support at birth and were excluded from the analysis. The demographics of the studied infants are displayed in Table 1. Overall, infants in the PPV group had significantly lower gestational age, birth weight, Apgar scores and received significantly less antenatal steroids (Table 1). Gender, rates of caesarean section and frequencies of delayed cord clamping were similar between groups. A total of 21,385 inflations and breaths (8,637 inflations and 12,748 breaths) were analyzed with a mean±SD of 408±159 per infant. 8953 (42%) inflations and breaths (3,471 inflations and 5,482 breaths) were excluded due to mask leaks of >30%. A total of 7,266 breaths in the CPAP group and 5,166 inflations in the PPV group were analyzed. Two infants in the CPAP group and eight infants in the PPV group required intubation in the DR. No infant received chest compression or epinephrine.

**Table 1:** Demographics of study infants.

	CPAP group (n = 31)	PPV group (n = 20)
**Birth weight (g)**	1763±487	1357±709^‡^
**Gestational age (weeks)**	31±2	29±3^‡^
Male*	11 (35%)	7 (35%)
Antenatal steroids*	27 (87%)	12 (60%)^‡^
Cesarean section*	22 (71%)	13 (65%)
**Apgar 1 minute^#^**	6 (4–8)	4 (2–6)^‡^
**Apgar 5 minutes^#^**	8 (7–8)	7 (5–8)^‡^
**Delayed cord clamping**	18 (58%)	11 (55%)

Data are presented as mean±SD unless indicated ^#^median (IQR), *n (%), ^‡^p<0.05.

### Exhaled CO_2_, V_T_, VCO_2_, and minute ventilation

**Figure 1:**
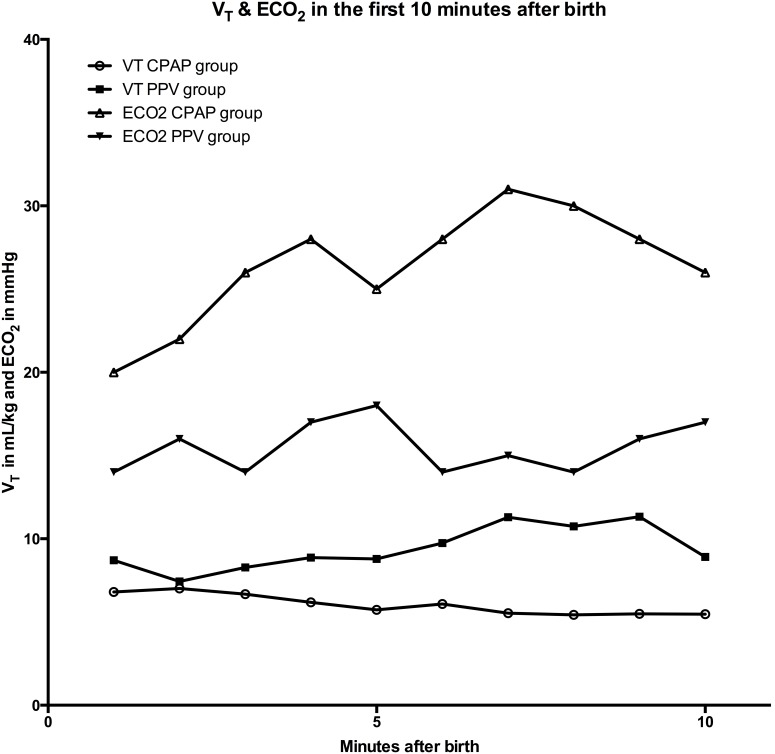
Tidal volume (V_T_) and exhaled CO_2_ (ECO_2_) during positive pressure ventilation (PPV) and in infants who are supported with continuous positive airway pressure (CPAP).

Throughout the first 10 minutes after birth, infants in the CPAP group had significantly higher ECO_2_ values (except at 5 and 10 minutes) compared to infants requiring PPV (Table 2, Figure 1). In addition, the V_T_ was significantly lower in the CPAP group compared to the PPV group over the first 10 minutes (except at two minutes) (Table 2, Figure 1). Minute ventilation was similar within the first five minutes, but at six to eight minutes it was significantly higher in infants supported with CPAP (Table 3). In the CPAP group VCO_2_ was significantly higher at 3, 5, and 8 minutes after birth (Table 3).

**Table 2:** Values of exhaled CO_2_ (ECO_2_) and tidal volume (V_T_) for infants supported with continuous positive airway pressure (+CPAP) and infants requiring positive pressure ventilation (+PPV).

	ECO_2_+CPAPmm Hg	ECO_2_+PPV mmHg	p-value	V_T_+CPAPmL/kg	V_T_+PPVmL/kg	p-value	p-value
**Minute 1**	21 (14–26)	12 (6–16)	0.015	6.8±3.6	8.7±3	0.034	0.074
**Minute 2**	23 (18–28)	16 (10–19)	0.004	7±3.7	7.4±2.4	0.441	0.165
**Minute 3**	27 (19–34)	17 (7–20)	0.0004	6.7±3.5	8.3±2.4	0.065	0.310
**Minute 4**	29 (19–35)	19 (6–22)	0.004	6.2±3.9	8.9±3.5	0.055	0.413
**Minute 5**	26 (17–34)	19 (6–27)	0.098	5.7±2.4	8.8±4.8	0.014	0.328
**Minute 6**	29 (20–36)	14 (10–18)	0.002	6.1±3.5	9.7±4.7	0.034	0.016
**Minute 7**	33 (23–37)	15 (13–19)	0.001	5.5±2.4	11.3±5.9	0.0002	0.003
**Minute 8**	32 (20–36)	11 (7–19)	0.003	5.4±2.1	10.7±6.8	0.0008	0.012
**Minute 9**	30 (23–34)	12 (10–20)	0.02	5.5±2.4	11.3±3.9	0.0001	0.304
**Minute 10**	26 (20–32)	14 (9–22)	0.059	5.5±3.3	8.9±3.3	0.031	0.150

Data are presented as median (IQR) for ECO_2_ and mean±SD for V_T._

**Table 3:** Values of minute ventilation (MV) and rate of CO2 elimination (VCO_2_) for infants supported with continuous positive airway pressure (+CPAP) and infants requiring positive pressure ventilation (+PPV).

	MV+CPAP mL/kg/min	MV+PPV mL/kg/min	p-value	VCO_2_+CPAP	VCO_2_+PPV	p-value
**Minute 1**	315 (157–416)	158 (81–323)	0.074	2.1 (0.4–4)	1.4 (1–2.8)	0.604
**Minute 2**	349 (257–430)	275 (205–356)	0.165	1.5 (0.4–6.3)	0.7 (0.1–2.1)	0.098
**Minute 3**	266 (160–394)	300 (233–454)	0.310	3.5 (2.2–8.9)	0.7 (0.2–1.7)	0.019
**Minute 4**	330 (166–491)	390 (228–566)	0.413	3.1 (1.7–5.8)	1.7 (0.3–2.9)	0.126
**Minute 5**	420 (183–510)	292 (176–408)	0.328	3.1 (0.4–9.8)	0.5 (0.1–1.3)	0.004
**Minute 6**	367 (258–574)	180 (126–325)	0.016	2.5 (1.4–3.7)	1.2 (0.1–3.6)	0.168
**Minute 7**	353 (238–575)	139 (111–255)	0.003	2.5 (0.6–3.7)	0.8 (0.3–1.8)	0.058
**Minute 8**	382 (268–600)	206 (116–357)	0.012	2.7 (0.9–7.7)	0.7 (0.3–1.6)	0.016
**Minute 9**	366 (213–534)	254 (180–350)	0.304	2.1 (1–5.2)	1.3 (0.4–1.6)	0.121
**Minute 10**	310 (213–500)	286 (68–331)	0.150	1.7 (0.9–4.1)	1.5 (0.3–3.3)	0.555

Data are presented as median (IQR) for MV and VCO_2._

### Respiratory rate, PEEP and PIP

In the CPAP group infants had a median (IQR) respiratory rate of 39 (32–50) breaths per min and in the PPV group 41 (30–49) inflations per min (p = 1.0). PEEP in the CPAP and PPV group was 6.2 (5–6.6) cm H_2_O and 6.5 (5.6–7.3) cm H_2_O (p = 1.0), respectively, and PIP in the PPV group was 24.7 (23.3–27.9) cm H_2_O.

### Heart rate, inspired oxygenation, oxygen saturation, crSO_2_ and blood pressure

**Figure 2:**
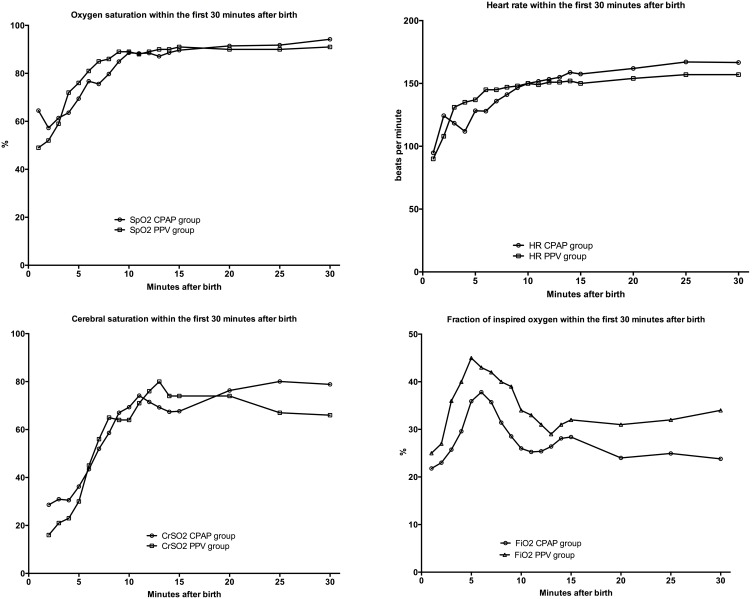
Changes in oxygen saturation (SpO_2_), heart rate (HR), fraction of inspired oxygen (FiO_2_) and cerebral oxygenation (rSO_2_) for infants supported with CPAP or PPV.

Changes in HR over the first 10 min after birth were similar between groups (Figure 2). More infants in the PPV group were started with respiratory support at 30% oxygen, and the fraction of inspired oxygen was significantly higher at two and three minutes after birth in the PPV group, compared to those of infants in the CPAP group (Figure 2). In addition, the fraction of inspired oxygen was significantly higher at 30 minutes after birth in the PPV group compared to the CPAP group with 0.34±0.15 and 0.24±0.60 (p = 0.033), respectively (Figure 2). The oxygen saturation during the first 30 min was similar except at one minute after birth oxygen saturation was significantly higher in the CPAP group compared to the PPV group 64±11% vs. 49±17%, p = 0.041, respectively (Figure 2). Cerebral oxygenation was significantly reduced only at 2 minutes after birth in infants receiving PPV 16±3% (vs. 29±12% in the CPAP group, p = 0.013), but at no other time point (Figure 2). Systolic, diastolic and mean blood pressure was significantly higher at 25 minutes after birth in infants receiving CPAP compared to PPV (p = 0.006). Mean±SD systolic, diastolic and mean blood pressure at 25 min in the CPAP group was 53/38 (37) compared to 44/27 (30) in the PPV group.

## Discussion

This observational study found, not surprisingly, that babies who require PPV after birth differ from those who breathe spontaneously and subsequently require CPAP. This study showed that preterm babies who require PPV immediately after birth follow a significantly different transitional process to those who breathe spontaneously using CPAP alone. The PPV group had lower ECO_2_ levels after birth, indicating lower lung aeration. We also observed a worse cerebral oxygenation. We infer that the lower ECO_2_ levels represent a delay in establishing FRC. This inference is supported by the observation that at 25 to 30 minutes of age, the PPV group had higher oxygen requirements (Figure 2). Interestingly, we also found that the infants in the PPV group had and or were resuscitated with high V_T_s to achieve similar lung aeration and ECO_2-_values at 10 min.

When infants fail to initiate spontaneous breathing immediately after birth, the international resuscitation guidelines recommend PPV [5]. In the DR, PPV is traditionally guided by changes in HR, however if HR does not increase chest rise should be assessed to gauge PPV [5]. This approach is supported by observational studies demonstrating that a rising HR is a very reliable surrogate for adequate ventilation [10], [11]. However, parameters to directly assess the efficiency of ventilation are lacking. Indeed, rising HR does not assess if the ventilation is excessive. *Hooper et al* recently described that ECO_2_ during PPV can be used to assess the degree of lung aeration and might have the potential to guide mask PPV in the DR [7]. In our current study ECO_2_ values were significantly higher in infants breathing spontaneously while supported by CPAP compared to infants requiring PPV (Table 2 & Figure 1). This is similar to previous observation suggesting that infants who are breathing spontaneously are able to aerate their lungs more effectively compared to infants requiring PPV [11]–[15]. It appears that lung aeration was achieved in two waves interrupted by a short plateau phase (Figure 1). We believe that infants establish FRC with the first wave pattern and minute ventilation with the second waves. Rising ECO_2_ and HR and higher VCO_2_ values identified the first phase and stabilizing ECO_2_, VCO_2_ and HR in the second phase (Table 2 & 3). Our data show that infants establish FRC within the first five minutes and continue gas exchange to achieve optimal oxygenation thereafter. Our data is supported by previously published data from asphyxiated term and preterm infants. These studies demonstrated a significant increase in VCO_2_ within the first 5 minutes and oxygen uptake within the first 8 minutes after birth [11]–[14]. Furthermore, infants in the CPAP group achieved lung aeration more effectively with lower V_T_ compared to infants in the PPV group suggesting that provision of PPV was associated with a slower FRC increase and not optimal to achieve adequate lung aeration (Figure 1) [11], [14] [15]. This is supported by recent studies suggesting that ECO_2_ values can be used to assess lung aeration [7], [15]. In addition, a recent randomized control trial using end-tidal CO_2_ to guide mask ventilation demonstrating that during CPAP end-tidal CO_2_ values were higher compared to PPV [16]. Further, our data suggest that despite higher and increasing V_T_s a slow improvement in lung aeration occurred and potentially improved ventilation strategies are required. The purpose of PPV is to establish a FRC and to facilitate gas exchange by delivering an adequate V_T_ without causing lung injury [6]. Resuscitators struggle to find a balance between adequate V_T_ delivery to achieve lung aeration and avoiding excessive V_T_ for lung injury [17]–[19]. In addition, most preterm infants breathe at birth and use several different breathing patterns to achieve lung aeration in the first minutes after birth [3], [20], [21]. Breathing and crying at birth results in partial lung aeration and therefore the use of fixed inflating pressures or the application of a sustained fixed inflation pressure in the presence of breathing could lead to considerable lung over-expansion [20], [22]. Our data suggest that if resuscitators are able to monitor both V_T_ and ECO_2_ they can adjust their respiratory support according to the individualized needs of the newborn infant. V_T_ may be misleading as it does not clarify whether FRC has been established – and even higher V_T_s do not necessarily imply aeration.

### Limitations

Although infants in the CPAP group were significantly older and heavier and therefore were likely to have more mature lungs, we believe that our results demonstrate that ECO_2_ can be used to guide respiratory support during neonatal resuscitation. Although, some of the infants enrolled in the current study were born at 23 weeks, particular in the CPAP group the infants were of relatively large gestational age. Translation of our results to infants of <28 week gestation should be done with caution. Furthermore, more infants in the PPV group were started on 30% oxygen (unit protocol), which potentially confounds the interpretation of SpO_2_, crSO_2_, HR measurements. In addition, we did not examine different breathing patterns to assess their impact on lung aeration, which is a limitation of the current study. A correlation of ECO_2_ values with the gold standard an arterial blood gas would have added to our manuscript, however this is not possible with the observed time frame. Although, end-tidal CO_2_ and arterial partial CO_2_ pressure are closely correlates in the neonatal intensive care unit [23], [24], these should not be extrapolated into the delivery room. ECO_2_ measurements are dependent on percentage of mask leak and airway obstruction, which are common problem during mask PPV [18], [25]. In the current study we excluded a total of 8953 (42%) inflations and breaths with mask leak >30% to avoid under-reporting of ECO_2_ values, which is a strength of the current study. The added dead space of the flow sensor potentially increases CO_2_ rebreathing, however the dead space is ∼1 mL and we did not observe any CO_2_ rebreathing. There are an increasing number of monitoring devices introduced into the delivery room [26]. However, it is important that human factors are considered when introducing new technologies, to avoid overwhelming the clinical team with the additional data [27], [28]. In addition, these new technologies have to be validated before they can become standard of care.

## Conclusions

Infants who breathe spontaneously have higher ECO_2_ values despite lower V_T_ throughout the first minutes after birth, suggesting improved lung aeration, compared to infants receiving PPV for respiratory support. Measuring ECO_2_ has the potential to assess lung aeration and to guide respiratory support after birth. Future delivery room studies should assess how newborn infants achieve lung aeration in detail and future trials should try to mimic the infant’s technique, which might have the potential to reduce short- and long-term outcomes in preterm infants.
